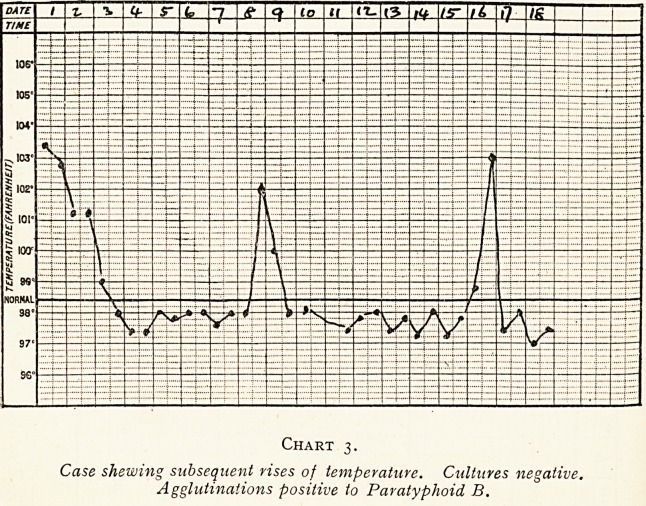# Notes on the Enteric Group of Diseases as Seen in an Isolation Hospital in France

**Published:** 1916-07

**Authors:** J. M. Fortescue-Brickdale


### NOTES ON THE ENTERIC GROUP OF DISEASES AS SEEN IN AN ISOLATION HOSPITAL IN FRANCE.

BY

J. M. Fortescue-Brickdale, M.A., M.D., M.R.C.P.,

Captain R.A.M.C. (T.).

In this paper I propose describing first the general arrangements of the hospital to which I was attached and the measures taken to isolate patients and prevent the spread of infection, and in the second part to give my own impressions as to the clinical features of the enteric group of cases, more especially with reference to Paratyphoid B.

The hospital consisted primarily of eight huts, each

120 feet by 15 feet, arranged in two sets on either side of a central path with their ends opposite to each other. Being somewhat narrow, only one row of beds could be placed in them, though a few beds could be put lengthwise against the opposite wall. Besides these huts we had eight "Armstrong Huts," made of canvas, holding two or three beds each; they are very nice in the summer when the side can be opened and an awning fitted to it, but we found them rather chilly and dark in the winter. We had also a number of bell tents, and later on the hospital was extended into hospital marquees, which were put up in a field below that on which the original huts were erected.

The various administrative buildings were constructed of corrugated iron and wood, and were placed on two adjacent sides of the square block of buildings formed by the wards.

The first thing found necessary was to divide the long huts into two parts, in order to isolate patients suffering from different diseases. We allotted wards or half wards to various infections as we thought they would be most likely to be required; but we soon found that the greater part of our wards would have to be given up to enteric, and that the arrangement of the remaining part would constantly require modification according to the prevalence of one or other of the infectious diseases. The Armstrong Huts were intended for officers, and were during the summer largely used for their accommodation, but some of them were generally kept for infections, such as scarlet fever or measles, of which we had only a few cases at a time, and were allotted to men. During the winter officers with enteric were placed in one of the half wards, and in the spring an officers' ward was built with separate rooms, holding two beds each, and kitchens, bathroom, etc., to itself. The bell tents were used for observation cases, but most of these were taken down in the winter, the Armstrong Huts being used instead.

The two main improvements made to the wards were the erection of a small sanitary annexe (6 feet by 6 feet) at each end of the long huts, and the construction inside the wards of a small sister's duty room and a scullery (8 feet by $5\frac{1}{2}$ feet), with a passage between them in the centre of the wards where the partition came.

A wooden reception-room and a large kitchen and stove for the service of the Armstrong Huts were also built during the autumn.

The regulations and arrangements for preventing cross infection and the spread of diseases from the hospital were briefly as follows.

All food for patients and orderlies was cooked and sent out from the hospital kitchen. The sisters and orderlies were not allowed to have any meals or food themselves in the wards, but took all their meals in their own respective messes. The orderlies on duty in the wards wore hospital blue, with their uniform caps, and both sisters and orderlies wore overalls. The orderlies changed their clothes when coming on and off duty in one of the sanitary annexes which was kept for the purpose. (The other sanitary annexe was used for keeping urine bottles and bed-pans.) Orderlies were not allowed to smoke on duty or to carry cigarettes behind their ears.

The kitchen had three storerooms, where meat, vegetables and milk were kept ; they were partly shut off from the main kitchen, and were covered in with muslin. Bread was kept in a fly-proof cupboard with gauze sides in the steward's store. Each patient had his own plate, bowl, spoon, and feeder, which were numbered and belonged to his bed.

The disposal of excreta and water used for washing patients was as follows. Bed-pans as soon as used were taken by the orderly to the destructor, covered by a cloth. The contents were mixed with sawdust and burnt in Horsefall destructor. The bed-pan was left with the sanitary orderly, who placed it in cresol till disinfected. It was then returned to the ward. Urine was carried in the bottle as soon as it was passed to the same building, and poured into a large boiler, and the bottle placed in a tub of cresol in a small adjoining room. The orderly carried back a clean bottle from the shelf in the same room. The urine after boiling was run by a pipe into a large soak pit. Water used for washing patients was also boiled in the same way before running into the soak pit. On the arrival of an ambulance with a suspected enteric case, the patient was taken on his stretcher to the reception hut, seen by the O.M.O., and his particulars taken by the N.C.O. on duty. The ambulance was sprayed with formalin, and a new stretcher and blankets given to the driver. The O.M.O. then sent the patient to the reception ward, the stretcher was sprayed, and the blankets and patient's kit taken to the steam disinfector.

At night the O.M.O. attended to the patient and if necessary called the M.O. who would have charge of the patient. During the day the M.O. saw the patient at once, and took a specimen of blood for the pathologist. In the course of the next twenty-four hours it was usually possible to say either on clinical or pathological grounds whether the patient clearly had an enteric infection, or whether he was a doubtful case who would require further watching and observation before a definite diagnosis could be made. He was then transferred either to an enteric ward or an observation ward; if the preliminary classification eventually turned out to be wrong he was again transferred to his proper place in the hospital. Those against whom no evidence of enteric infection could be brought either by the clinician or the pathologist found their way eventually into a special ward reserved for "wash-outs," and were labelled influenza or myalgia or P.U.O. according to circumstances. These patients, when well, were sent to their base depots or to the convalescent depot at the discretion of the M.O. The pathological investigations included at least one blood culture, three agglutination tests against Typhoid, Paratyphoid A. and Paratyphoid B., and three examinations of the fæces and urine for organisms. When fit to walk about and take light exercise, all enterics were evacuated to England, and notes of the case typed on the back of their transfer certificates signed by the C.O.

These measures were very successful. I can remember only two orderlies who contracted enteric, and though we had some cases of double infection among patients, I can remember only one in which we certainly thought that the second infection might have been acquired in the hospital. Of course, constant supervision of the orderlies was necessary: lectures were arranged for them and given by the M.O.'s which dealt with infectious diseases and sanitation. If they were caught disobeying rules, the matter was reported to the C.O., and punishment inflicted when it seemed necessary.

The "Enteric Group" is taken to include Typhoid, Paratyphoid A. and Paratyphoid B. and the term "enteric" is not used as synonymous with typhoid, but as designating the group. In making returns, if the bacteriological evidence was not sufficient to identify a case, but clinically the patient appeared to have one of the three diseases, we were instructed to diagnose it as "Enteric Group." The proportions in which the three diseases occurred were roughly 10 per cent. Typhoid, 4 per cent. Paratyphoid A., and 81 per cent. Paratyphoid B., the remaining 5 per cent. being "Enteric Group."

It is, of course, impossible to diagnose clinically the presence of a particular causal organism in any disease. This can only be done by means of bacteriological investigation.

But just as diphtheria can be in practice distinguished from other forms of tonsillitis in a number of cases, so it is occasionally possible to give an accurate guess as to which of the enteric groups a patient belongs. But, as a rule, this can be done only when the patient has been under observation for some time, and the general features and course of the attack have been noted. It is more possible to distinguish clinically between typhoid and the paratyphoids than between Paratyphoid A. and B., though in some cases the former present suggestive features. Practically, however, the diagnosis always rests with the bacteriologist, and no clinical diagnosis is accepted other than that of "Enteric Group." I propose now to set down some rough notes on the symptoms and course of the paratyphoid fevers as I saw them.

1. *Onset.*—The mode of onset appears to have some relation to the severity of the attack. Three types may be distinguished. Those in which the onset is quite abrupt; the patient goes to bed feeling quite well, and wakes up next morning unable to go on duty. In the next group there is a week or less of prodromal symptoms, not very serious or definite in character. In the third these prodromal symptoms are noted for a longer time, and the onset is more gradual. A larger proportion of severe cases were found to follow on this third type of onset, and the smallest proportion to follow the first type of onset.

Initial symptoms are many in number, but by far the most frequent is headache. Diarrhœa and shivering are not uncommon, and sometimes pain in the back and abdomen; vomiting and cough are less common. Fainting, nasal catarrh, laryngitis of mild type, herpes labialis and conjunctivitis are occasionally seen. Epistaxis is not a frequent initial symptom, but is not uncommon at some period in the attack.

2. *Pyrexia.*—In our cases of Paratyphoid B. we found about 80 per cent. with ten days' fever or more, and 20 per cent. with under ten days' fever. In analysing the former group I was able, I thought, to distinguish the following types :—

(*a*) Simple continued fever, often with a tendency to remission, but with no very characteristic curve.

(*b*) Intermittent type, the intermissions usually occurring towards the end of the attack, but sometimes at the beginning, or more or less throughout. Nearly half the cases showed this type of fever, which is illustrated by Chart 1.

(*c*) Undulant type. In half these cases the notes showed that there were more or less well-marked pulmonary symptoms, but beyond that the type of fever could not be correlated to any other features in the attack. (Chart 2).

(*d*) Low, irregular fever. In this type, often after a few days' high fever, the temperature fell to normal or subnormal in the morning, but rose to 99 per cent. or 100 per cent. F.

CHART 1.

*Intermittent type of pyrexia. Paratyphoid B. isolated from urine 18th day. Agglutinations positive to Paratyphoid B.*

Chart 2.

*Undulant type of fever. Paratyphoid B. isolated from fæces on 8th day of disease. Agglutinations positive to paratyphoid B.*

in the evening, and sometimes a little higher. The attack was often prolonged.

(*e*) In a certain number of cases, a week or so after the temperature had fallen there was a sudden rise of temperature lasting about twenty-four hours, usually accompanied by headache or general pains. These rises were sometimes repeated several times. We often found the spleen tender, but could not detect any tenderness over the gall bladder, which has been noted by other observers. The attacks are not serious, and the patient is generally well the next day. (Chart 3).

3. *Facies and Constitutional Symptoms.*—The patient's appearance is often quite without any diagnostic significance but in some cases he has a peculiar heavy, "bloated," coarse appearance, which is rather suggestive. The constitutional disturbance may be considerable, but on the whole in paratyphoid the patient is not so ill as in typhoid, having regard

CHART 3.

*Case shewing subsequent rises of temperature. Cultures negative. Agglutinations positive to Paratyphoid B.*

to the duration and height of the fever. Convalescence as a rule is much more rapid and satisfactory in paratyphoid.

4. *Skin.*—The eruption is common but not constant. Its chief characteristic is its polymorphism, spots of many shapes and sizes being observed on the same patient. It is seldom earlier than the fourth day, but the crops may continue to come out for several weeks, and even after the temperature has become normal fresh spots may be observed. Jaundice was observed in a few cases.

5. The spleen is nearly always tender, and can often be felt. It is usually soft. Increase of splenic dulness always seems to me an unreliable sign.

6. Severe complications were unusual in our series. We had a few cases of hemorrhage, but none were very severe. We lost a few cases from perforation, and saved none by operation. Thrombosis, generally of the femoral vein, was not very uncommon, and one case had symptoms of pulmonary embolism from which he recovered. Parotitis and orchitis were observed, and sometimes go on to suppuration. We had one case of meningitis, one or two of boils, one of hæmaturia, and one of herpes zoster.

7. Relapses were infrequent, but sometimes more severe than the original attack.

8. As regards treatment, we relied on dieting and nursing; we tried a few cases with typhoid vaccine, but we did not think any good ensued. The mortality among the paratyphoids was very low, though I cannot now state the exact figure. Though many patients had a severe illness, but a small number appeared to be in actual danger.

9. Anomalous cases occurred in which the bacteriological evidence, including three agglutination tests, was negative, but which presented symptoms of enteric infection, such as spots or an enlarged spleen. Moreover, some cases in which the organism was isolated from the urine or fæces, failed to show the presence of specific agglutinins in their blood. In other instances in which the bacteriological evidence was complete, the duration of the fever was so short that they might easily have been passed over or diagnosed as "febricula."

## Figures and Tables

**Chart 1. f1:**
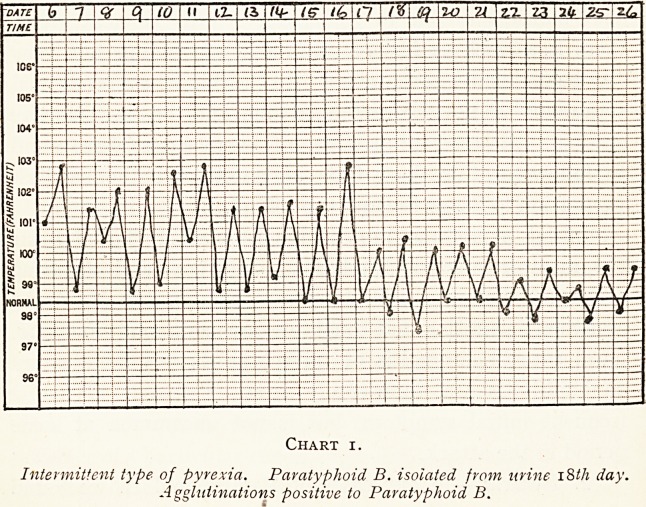


**Chart 2. f2:**
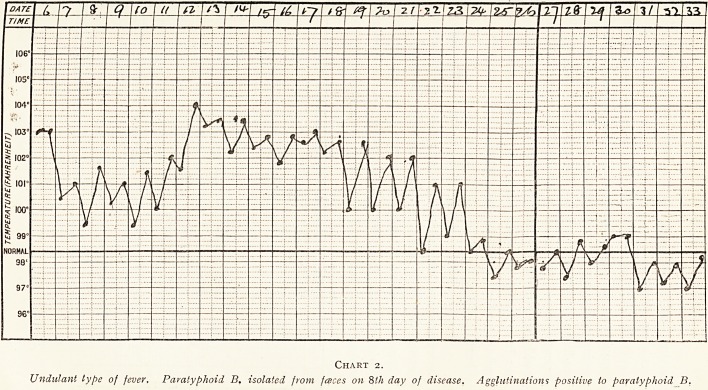


**Chart 3. f3:**